# Production of Long-Fiber Pulp from Enset Plant Residues by Soda Pulping

**DOI:** 10.3390/molecules29204874

**Published:** 2024-10-14

**Authors:** Hanna Berhanu Lemma, Friedrich Steffen, Abubeker Yimam Ali, Bodo Saake

**Affiliations:** 1 Institute of Wood Science, University of Hamburg, Haidkrugsweg 1, 22885 Barsbuettel-Willinghusen, Germany; bodo.saake@uni-hamburg.de; 2 Addis Adaba Institute of Technology, Addis Adaba University, 2QR7+584, King George VI St, Addis Ababa 1000, Ethiopia; abubeker.yimam@aau.edu.et

**Keywords:** non-wood, alternative fiber sources, Enset fiber, soda pulping, refining, papermaking, paper strength, fiber morphology, fiber length

## Abstract

This paper evaluates the modification of fiber morphology and the strength property development of paper from Enset fiber as a function of soda pulping conditions and refining energy. Soda pulping was conducted at pulping temperatures between 160 and 180 °C. The NaOH charge was 16, 20, and 24% based on the initial raw material. The beating of pulp was conducted using a Jokro mill. The refining of pulp was conducted in a laboratory refiner at different refining intensities. The mild Jokro mill beating was not effective on Enset fiber pulp. On the other hand, the laboratory refiner effectively refined the pulp. The fiber morphology was altered in the way of improving the paper formation and strength. The beating degree of the pulp was increased to about 49 °SR. The tensile index was enhanced to around 80 Nm/g using a refining energy input of 250 kwh/t. From the results, it can be concluded that Enset fiber pulp is suitable for packaging papers due to its high strength level. On the other hand, Enset fiber can be a potential raw material for specialty papers like filter paper and tea bags because of its high porosity.

## 1. Introduction

Even if digitalization has led to a drastic decrease in graphic paper demand, the paper and pulp industry is still growing as packaging and tissue papers are filling the shrinking graphic paper market share gap. Various non-wood plants and agricultural residues are already being used to satisfy the pulp and paper fiber industry demand.

Enset plant fiber is one of the readily available agricultural residues in southern and southwestern parts of Ethiopia, with increasing interest being generated in natural fiber research. It is a compact yellowish-white cellular bunch of predominantly long cells (fibers), vessels, and parenchyma. The length of the cellular fiber obtained after dissociation of the bundle varied from 2.6 to 11.4 mm, and it had a width varying from 15.3 to 18.1 μm, a lumen width varying from 7.0 to 11.2 μm, and a cell wall thickness varying from 2.3 to 4.6 μm. The fiber comprises a high proportion of holocellulose (87.5%), of which 60% is cellulose. The amount of lignin is relatively low (10.5%). However, a strong delignification condition may be needed to delignify the fiber effectively due to the lower S/G ratio in the lignin monomeric composition (H:G:S, 1:0.7:0.8). The mineral components of the fiber are dominated by potassium, whereas, in the micronutrient, iron is present in significant amounts. Due to its promising characteristics, Enset fiber can be considered a good candidate as a long-fiber source in the pulp and paper industry. Berhanu et al. [1] reported a pulp yield of around 70%, with a low kappa number of around five and excellent sheet paper strength properties during the ethanol alkaline pulping of Enset fiber without further bleaching and a pulp refining stage. The major influencing factor in the pulping experiment was the NaOH charge, followed by temperature, and reaction time had little effect on the resulting pulp [1,2].

Soda pulping is the preferred process for the pulping of annual plants [3]. It uses sodium hydroxide (NaOH) as an active chemical under high pressure and temperatures to allow fiber swelling, and it increases the better penetration of the cooking chemicals [4]. Besides delignification, the peeling reaction of glucosidic bonds could be of significant concern during soda pulping since there are considerable yield losses due to carbohydrate degradation by the peeling reaction [3]. Previous studies have shown that the amounts of dissolved carbohydrates in the cooking liquor increase rapidly during the initial delignification of the cooking. The dissolution of carbohydrates then decreases during the bulk delignification phase, growing again in the final delignification [3]. Thus, to reduce the degradation of carbohydrates and promote delignification, a proper alkali charge and reaction temperature should be selected.

The other vital phase in the paper industries is pulp refining or pulp beating. Pulp refining is a mechanical treatment step to improve the paper formation and paper mechanical properties. Compressive, shear, and bending forces are randomly and repeatedly undertaken on the fibers during refining. This mechanical action reduces fiber stiffness and increases fiber flexibility. Moreover, the surfaces of the fibers are modified so it improves the fiber-to-fiber bonds in the paper [5,6,7,8,9]. Refining causes significant changes in the fiber structure, such as cutting, the swelling of fiber walls, external and internal fibrillation, increased flexibility, and curling [10]. Those refining effects lead to changes in density, porosity, formation, and other essential paper properties [5,11].

Refining also lowers the pulp drainage capability. That means it reduces the paper production rate and increases the energy consumption. One of the essential features of pulp is the beating degree. It is the susceptibility of the pulp to refining that is key as refining energy is required to reach a specific water retention value or tensile index. The mechanical action of refining causes cavity formations and delamination of the fiber wall, which allows the water to penetrate through the fiber wall, resulting in a more significant swelling potential [11]. The water makes the fiber much more flexible and relevant to breaking hydrogen bonds. Whenever the fibers are thoroughly wetted, refining disrupts the crystallinity of fibers, and more water can be added to the cellulose fibers. This phenomenon is known as internal fibrillation, and it is among the main reasons for improving the strength properties of paper.

External fibrillation is, on the other hand, when fibrils are peeled off from the surface of the fiber surface while still attached. Fibrillation exposes the cellulose fibers and increases the fiber surface area to improve the fiber-to-fiber bonding of the final sheet. The fibrils enhance the bonding between fibers through mechanisms such as mechanical interlocking, leading to paper with higher tensile strength. Furthermore, the surface area of the fiber increases and becomes more flexible to fit in around each other. These increase the bonding surface area and lead to a denser sheet [7]. However, due to the reduction in the strength of individual fibers, external fibrillation can negatively influence the tear strength [11].

The fibers also change their geometric shape and fibril alignment along their length, and the fibers flatten or collapse. Fiber curl and kinks are induced or straightened. Moreover, little dislocations and compressions are generated or diminished in the fiber [7].

Fiber cutting and breakage also occur during refining. This contributes to the changes in fiber length distribution and decreases in the mean fiber length. In addition, the proportion of fines might be increased. Fines have pros and cons regarding the strength of the final paper, and they are conditional to their shape and type [11]. The primary fines are present in the pulp before refining. They have an adverse effect on the strength of the paper. Secondary fines are created during refining, and they are mainly composed of fibrils. They improve the strength of the paper by increasing the effective contact area of the bonded fibers, which can help establish contact between close fibers [6]. Generally, some fiber morphological properties are modified in a way to improve paper properties, but others deteriorate with refining. All these changes that co-occur during refining are irreversible. The morphology of the fiber and the refining conditions governs the extent of the changes in the pulp. The refining conditions are the design of the refining equipment and its operating variables, such as the consistency, intensity, and refining time [7,12].

The refining equipment can be conical or disk-type refiners with a parallel flow to the bar crossings. Refining power is a measure of the power input to the motors of the refiner based on the amount of pulp processed. It is an indirect measure of the energy used in cutting and fibrillating the pulp fibers, although these processes consume only a small percentage of the power. These values are essential in the design and economic calculations. The specific refining energy is commonly expressed in kilowatt hours per ton of pulp processed (kWh/t) [7,13].

The different pulp originating from various fiber sources have varying fiber lengths, cell wall thicknesses, and widths to their central lumen. They respond accordingly to a given type of refining and set of refining conditions, and not all fibers must receive the same treatment. Some fibers are fibrillated more by one kind of beating than another. Thus, the right refining type and intensity should be selected based on the type of fiber and desired paper properties.

In the present paper, the effect of soda pulping parameters and the type and severity of refining on the morphology of the fiber and the physical paper properties were studied.

## 2. Results and Discussion

### 2.1. Laboratory-Scale Soda Pulping and Jokro Mill Beating

**Figure 1:**
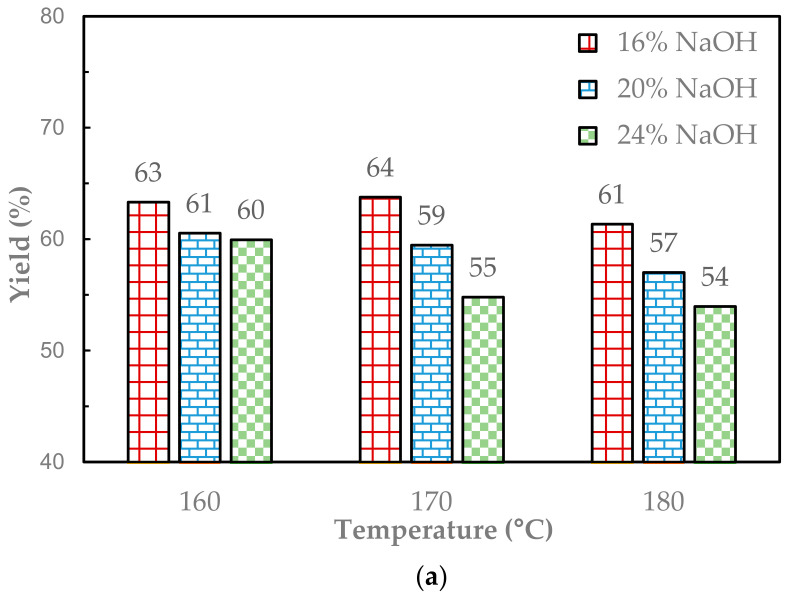

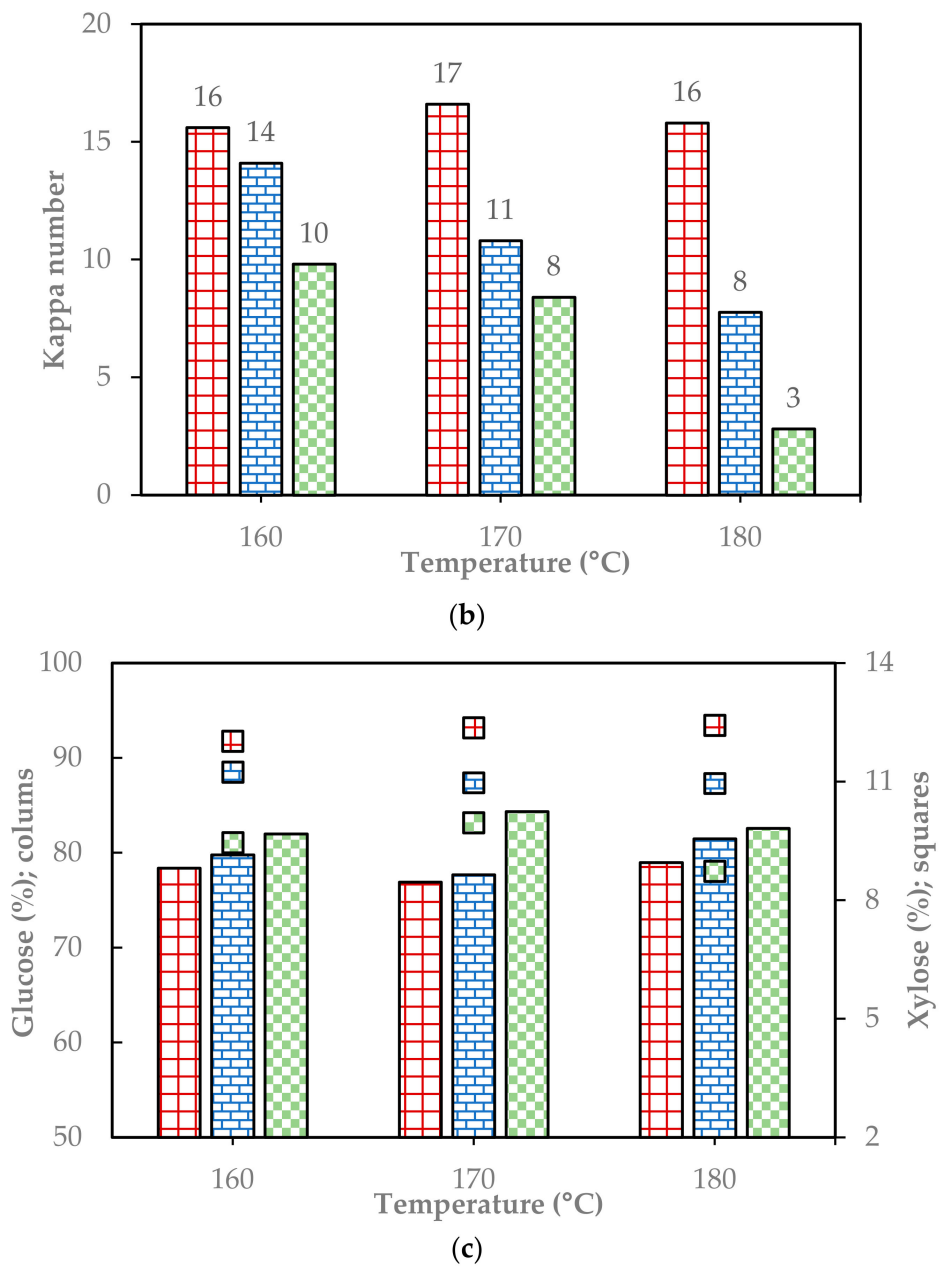
The pulp yield (**a**), kappa number (**b**), and carbohydrate composition (**c**) of the soda pulps from the pulping of Enset fiber at different temperatures and different NaOH charges.

In the first series of experiments, the soda pulping of Enset fiber was investigated using a laboratory-scale rotating digester. The pulping temperature varied between 160 and 180 °C. The NaOH charge was 16%, 20%, and 24% based on oven-dry raw material. All other pulping parameters were kept constant. The pulp yield, the kappa number, and the carbohydrate composition of the soda pulps from the pulping in the laboratory-scale digester are presented in Figure 1.

The pulp yield and the kappa number dropped with the pulping condition severity, as shown in Figure 1a,b. This was due to the degradation and dissolution of mainly lignin, as expressed in the decrease in kappa number. However, under such strong alkaline conditions, the degradation and dissolution of cellulose and hemicellulose also occurred. The latter can be clearly seen from the graph in Figure 1c, where the xylose content of the pulp was at the lowest level when the highest NaOH charge of 24% was applied.

The NaOH charge was the dominant factor in the soda pulping of Enset fiber compared to the pulping temperature [1]. The effect of temperature was found to be statistically insignificant (*p*-value < 0.08), whereas the NaOH charge was found to be statistically significant (*p*-value < 0.01). As can be clearly seen in Figure 1a, the effect of temperature on the pulp yield was not significant for pulping at a lower NaOH charge and was significant for pulping at a higher NaOH charge. The same scenario was obtained in the statistically significant result in the kappa number and carbohydrate composition (Table S1).

Berhanu et al. [1] reported a similar situation in the ethanol alkaline pulping of Enset fiber, where the alkali charge was the dominant factor compared to other pulping parameters. This might be because the peeling reaction can occur whenever the alkaline liquor is in contact with the lignocellulosic material, even at low temperatures, as stated by Ek et al. [3].

**Figure 2:**
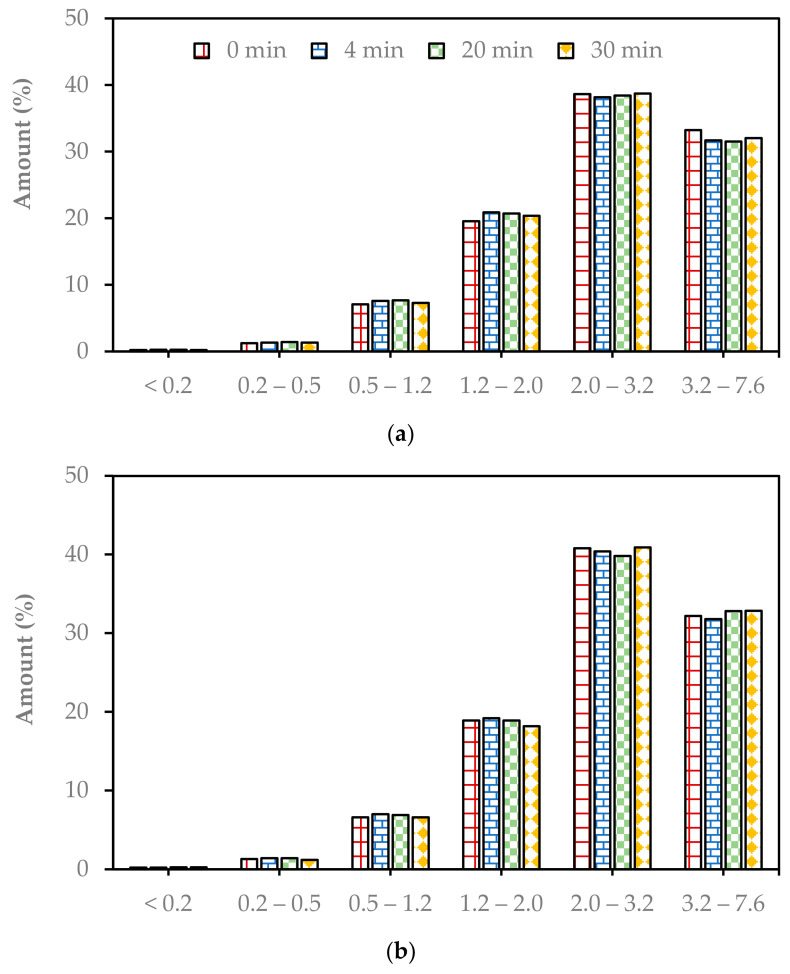

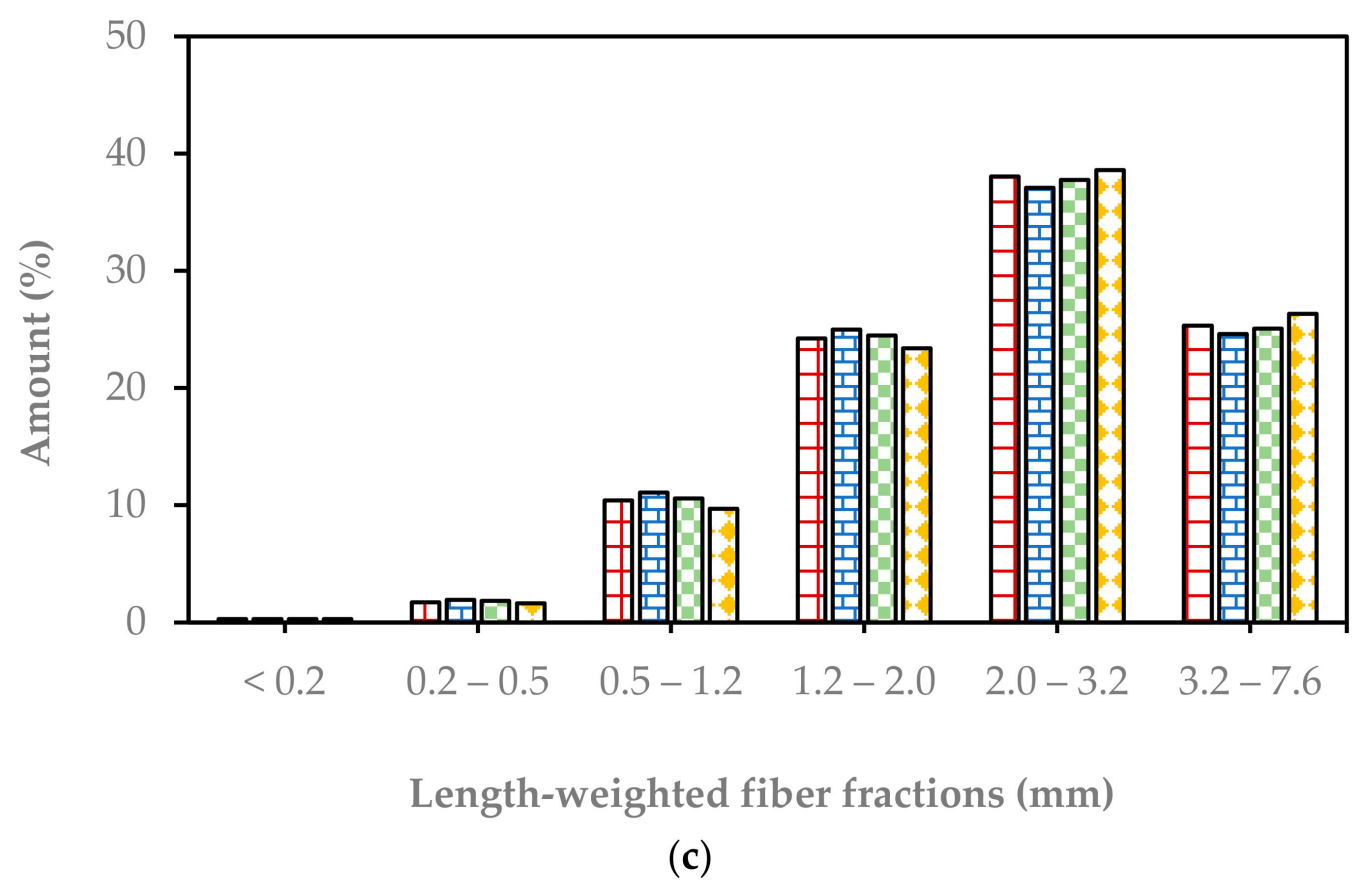
Fiber length distribution data for the pulps made at 160 °C (**a**), 170 °C (**b**), and 180 °C (**c**) at a 16% NaOH charge at beating times of 0–30 min.

The fiber length distribution and fiber morphology of the selected pulp was measured before and after beating in a Jokro mill for 4 min, 20 min, and 30 min. The fiber length distribution data for pulps made at 160 °C, 170 °C, and 180 °C at a 16% NaOH charge are presented in Figure 2.

The pulp comprised fine particles and short and long fibers. The average fiber length of the Enset fiber soda pulp was 2.8 mm, which was not altered due to Jokro mill beating, as can be seen in Table 1. The soda pulp from Enset fiber had a longer average fiber length and lower average fiber width than most agricultural residues, such as wheat straw and rye straw [14]. These morphological properties resulted in a higher slenderness ratio of Enset soda pulp, making the fiber a more suitable raw material for paper industries with higher strength properties [14,15]. The content of primary fiber fines in the Enset fiber was also lower than that in the wheat straw and rye straw. The fines present in the pulp before refining adversely affect the strength properties of the final paper [11].

The long fibers (>2 mm) were 63% of the pulp, and the fine particles (<0.2 mm) were less than 2% of the pulp. Figure 2 shows that was no significant change in the fiber length distribution due to Jokro mill beating. Statistical analysis also demonstrated the ineffectiveness of Jokro mill for Enset fiber pulp (Table S2). In addition to the fiber length, the other fiber morphological properties were not altered meaningfully and significantly due to Jokro mill beating, as shown in Table 1.

The strength property of the paper sheets was measured for all nine samples with a 15 L rotary digester to see the possible effect of Jokro milling at different beating times. The strength properties of the pulps are presented in Figure S1 (Supplementary Materials).

The Schopper-Riegler value ranged from 17 to 36 °SR without any defined correlation with the beating time or pulping condition. The tensile and burst indexes of the unbeaten pulp produced by 16% NaOH were much higher than the other two unbeaten pulp produced by a 20% and 24% NaOH charge. In contrast, the tear index was much lower for the unbeaten soda pulp produced by milder pulping conditions, as shown in Figure S1. The paper from Enset fiber has higher strength properties than Bagasse, wheat straw, and rye straw. As explained earlier, this can be due to the superiority of the morphological property of Enset fibers [16,17,18]. Unlike wheat straw, the beating time, beating degree, and strength properties of Enset fiber do not correlate during Jokro mill beating [17]. The porosity (gurley) and specific volume of unbeaten pulp produced by milder conditions were much lower than those produced by severe conditions (Figure S1). The porosity of the hand sheet from unbeaten Enset fiber pulp was much higher than that of the hand sheet from wheat straw and bagasse. This can be explained by its lower amount of primary fine particles in the pulp, lower fibrillation, and longer fiber length [17,18].

Moreover, ununiform fiber distribution in the formed paper sheet was visible to the naked eye, and some of the fibers were even observable. This indicates that the mild Jokro mill treatment is unsuitable for Enset fiber. Therefore, a Voith LR 40 refiner had to be used to see the full development of the strength potential of Enset fiber. For this experiment, larger quantities of pulp were required. Upscaled pulping was performed in a large paddle digester simulating the horizontal tube digester usually applied for the pulping of the annual plants in the industries.

### 2.2. Pilot-Scale Soda Pulping

**Figure 3:**
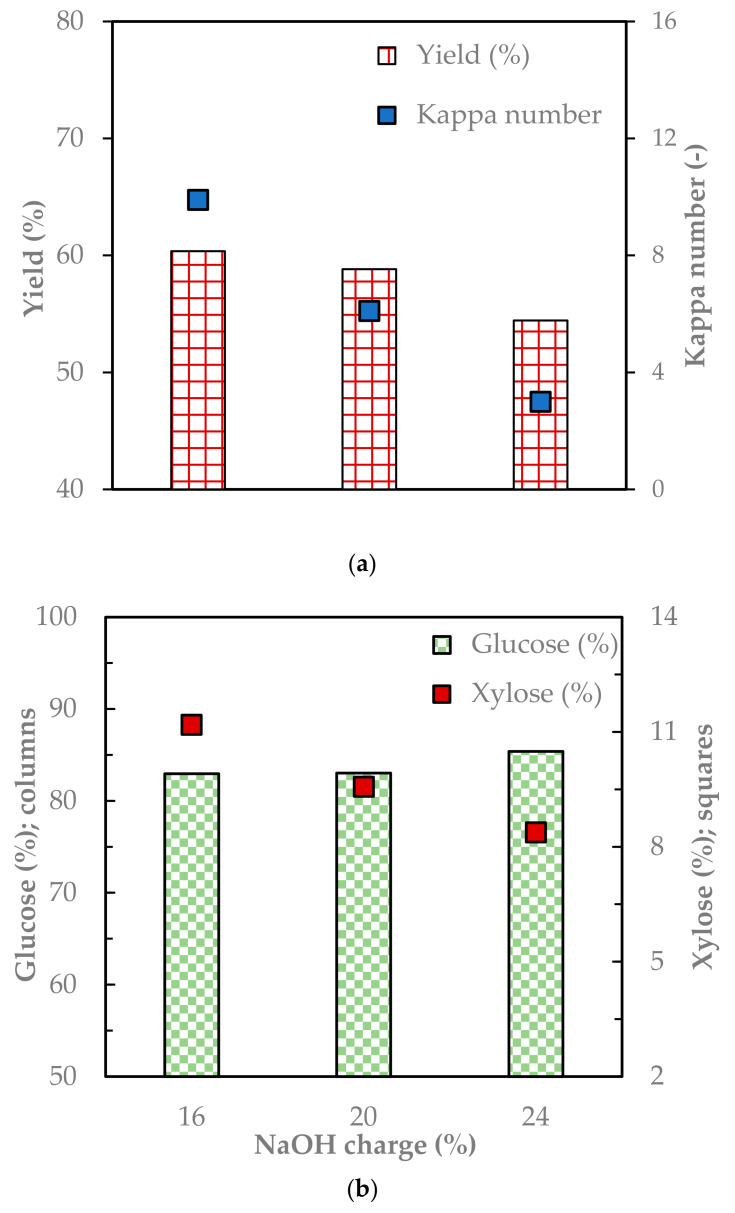
The pulp yield, kappa number (**a**), and carbohydrate composition (**b**) of the soda pulps from pulping at different NaOH charges using a paddle digester.

Figure 3 shows the yield, kappa number, and carbohydrate composition of the pulps from a paddle digester compared to a small electrical digester at the same cooking conditions. The pulp yield from the paddle digester was 60% for mild and 54% for severe pulping, and the corresponding kappa numbers were 9.9 and 3, respectively (Figure 3a,b). On the other hand, the yield was 65% and 55%, and the kappa number was 16.6 and 8.4 for the small electrical digester at the same pulping conditions. The type of digester did not significantly affect the pulp yield (*p* value < 0.26). On the contrary, the kappa number and the total lignin content were much lower for the paddle digester (Figure 3b). The total lignin content for the pulp was between 1.6% and 1% for the paddle cooker and 7% to 1.6% for the electrical digester at the same cooking conditions. The large-scale pulping in the paddle cooker was effective in selective delignification (*p* value < 0.05) due to the high mixing efficiency in the paddle reactor.

The carbohydrate composition of the pulp was evaluated because it affects the final characteristics of the paper. Paper with higher hemicellulose and cellulose content show higher tensile strength because they are responsible for the fiber-to-fiber bonding in the paper. In addition, the bonding is improved by hemicellulose through its ability to hold water during processing [19]. On the other hand, lignin prevents the formation of fiber-to-fiber bonds in paper and reduces paper strength [12]. The result shows that the paddle reactor removed xylose more effectively than the small electrical digester. The amount of xylose was from 11% to 8.4% for pulps from the paddle reactor, whereas it was 12% to 10% in the electrical digester at the same cooking conditions. The reactor type significantly affected the removal of xylose in the pulp (*p*-value < 0.05). On the other hand, the glucose proportion was comparable to the pulp from a small electrical reactor.

### 2.3. Refining and Fiber Morphology

**Figure 4:**
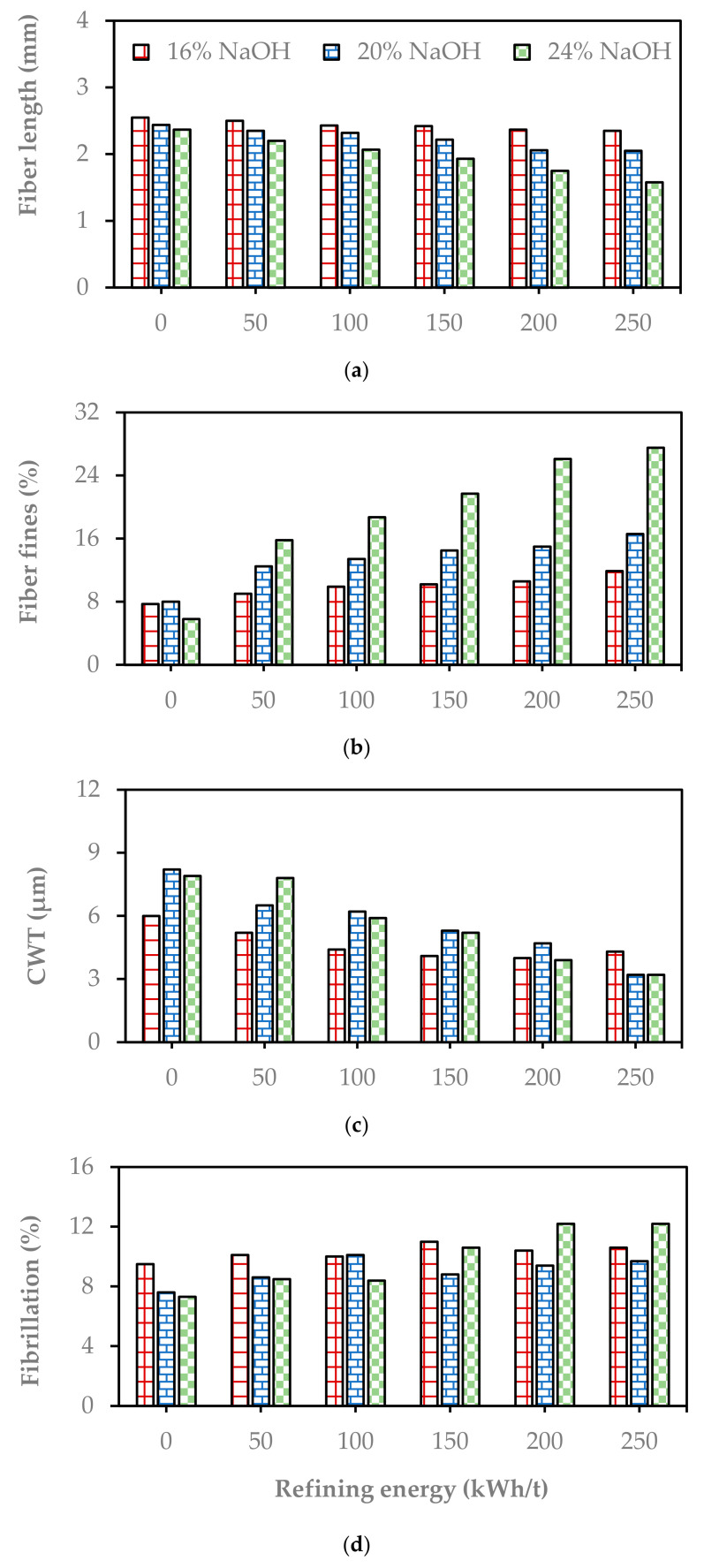
The average fiber length (**a**), fiber fines (**b**), CWT (**c**), and fibrillation (**d**) of the soda pulps from pulping at different NaOH charges depending on the refining energy.

The pulp from the paddle digester was refined in a laboratory refiner (Voith LR 40) at different refining energy levels up to 250 kwh/t. The average fiber length of the pulp decreased with the pulping condition severity and refining energy, as shown in Figure 4a. The difference between the average fiber length of the unrefined pulp in mild and severe conditions was 0.2 mm. The variation became 0.8 mm at the refining energy level of 250 kwh/t, as shown in Figure 4a. The result clearly indicates that the pulping severity affects the average fiber length and makes the fiber susceptible to refining.

On the other hand, the fine fraction in the pulp increased with increasing pulping severity and refining energy (Figure 4b). It is clear from the slope of the graphs in Figure 4b that the increasing rate of the fine fraction in severe conditions was higher than the other two pulps due to the weakness of the fiber in resisting the refining effect.

**Figure 5:**
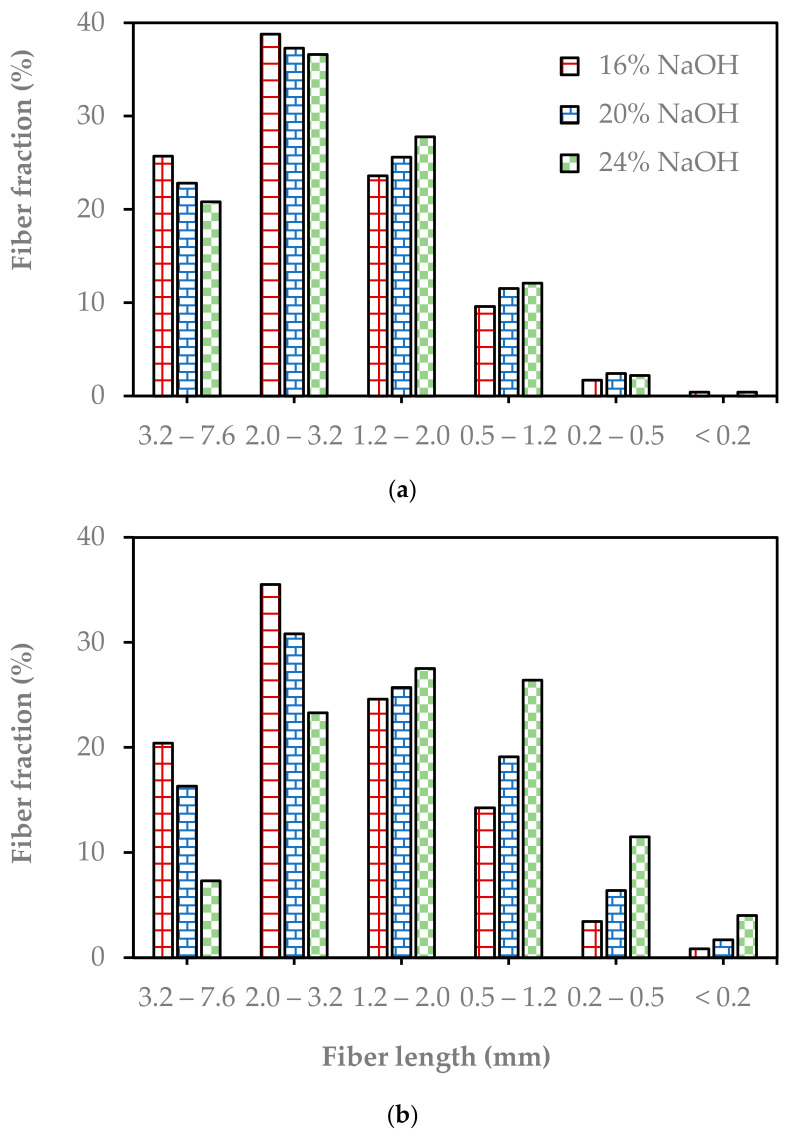
Fiber length distribution of the soda pulps from pulping at different NaOH charges depending on the refining energy: (**a**) 0 kWh/t and (**b**) 250 kWh/t.

**Figure 6:**
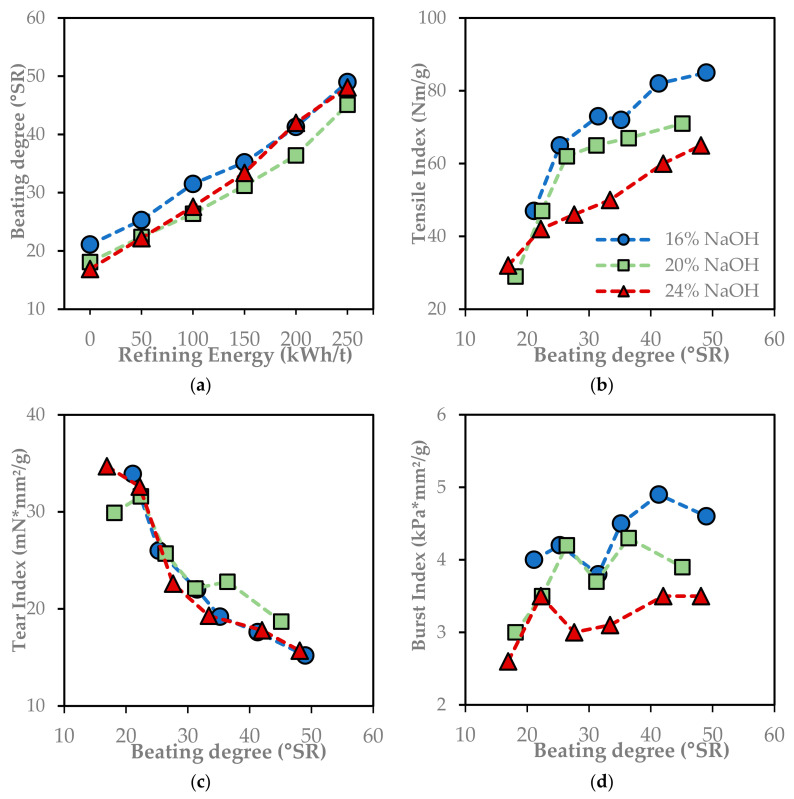
The beating degree (**a**) and strength properties of the soda pulps from pulping at different NaOH charges depending on the refining energy: (**b**) tensile index; (**c**) tear index; and (**d**) burst index.

Figure 5 shows the impact of the pulping severity and refining intensity on the fiber length distribution. The proportion of long fiber (>2 mm) decreased while the proportion of short fiber (<1.2 mm) increased with increasing soda pulping conditions and pulp refining energy. The longest fiber fractions (>3.2 mm) of the unrefined pulp decreased from 25.7% to 20.8% with pulping severity. The proportion of long fiber was also remarkably reduced with refining energy. It declined at a higher rate for the pulp produced with severe pulping. It decreased by 5.3%, 6.5%, and 13.5% at 16%, 20%, and 24% NaOH charges, respectively. On the other hand, the fine fraction (<0.5 mm) increased by 0.43%, 1.2%, and 3.6%, respectively (Figure 6). The weakness of the fiber due to severe pulping conditions facilitated the formation of fine particles. Statistical analysis of the fiber length distribution proved that the fiber length distributions depended on both pulping and refining conditions (Table S2). Moreover, the pulping severity and refining condition interaction significantly affected the fiber length distribution. The Enset fiber had a wide range of fiber length distribution compared to other agricultural residues for instant EFB fiber, which only ranged from 0.2 mm to 1.6 mm, and the wheat straw ranged from 0.1 to 1.7 mm [20].

The refining intensity indirectly affects the fiber width, fiber surface area (CSA), and cell wall thickness (CWT). As reported by Berhanu et al. [2], the cell wall thickness of the raw Enset fiber was in the range of 2.3 to 4 µm. The cell wall thickness increased to 6 µm for pulping when using a low NaOH charge of 16% and to 8 µm when using a high NaOH charge of 24% (Figure 4c). The increase in CWT was due to the swelling effect of the increasing NaOH charge on the fiber cell. The fiber width and surface area decreased with refining due to the deformation of the cell wall and the lumen. They decreased with refining due to the pressing of two opposite walls and a collapsing of the fibers, or due to the lumen being eliminated partly or entirely. The other crucial parameter was fibrillation, which makes the fiber flexible and ready to form fiber-to-fiber bonds in the paper. This improves paper formation, where the fibrillation of the pulp increases with refining (Figure 4d). The curl index decreased with increasing refining energy. Fiber curls are directly related to the tensile index and influence the stretchability of a paper, so they are more critical on sack paper [12].

### 2.4. Refining and Paper Strength Property

The beating degree and paper strength properties strongly depend on the original structure of the fiber and the morphological modification of the fibers during refining [12]. In addition to various interrelated fiber properties, the strength of the hand sheet paper is also affected by pulping techniques. Therefore, it is not easy to find correlations that justify every scenario [21]. The primary targets of refining are to improve the strength and formation of paper. The extent of refining is monitored by measuring the beating degree (Shopper Riegler SR° pulp freeness) and the strength properties of hand sheets [7].

The refining of Enset fiber pulp using a Voith refiner showed apparent enhancement in SR° and in the strength properties of the paper, as shown in Figure 6a. The °SR value of unrefined Enset fiber pulp at different NaOH charges was 17 to 21. The °SR linearly increased with increasing refining energy. At the refining energy of 250 kWh/t, the °SR reached 45 to 49. The increase in °SR value was related to the surface conditions and swelling of the fibers.

Moreover, the internal and external fibrillation and fine formation was the main reason for the increment in the Shopper Riegler value [22]. Olejnik et al. [5] also reported an increment in the water retention value of fiber with increasing refining energy. Compared to bagasse and wheat straw, Enset fibers are resistant to refining. The SR° value of wheat straw has already reached 55 and bagasse soda pulp has reached 75 at 120 kwh/t and 200 kwh/t refining, respectively [17,18].

Paper sheets were then formed using unrefined and refined pulps to analyze the improvement of the strength properties of the paper. Figure 6b–d shows the strength property of the paper plotted against the beating degree.

The tensile index and burst index of the paper sheet increased with increasing SR°, while the tear index decreased (Figure 6b–d). The tensile index was improved through increasing bonding potential due to fibrillation and fiber flexibility [23]. The fibrillation of the individual fiber sourced a better fiber-to-fiber contact area. The reduction in average fiber length negatively affected the tensile strength of the paper. However, the reduction in fiber width and cell wall thickness compensates for the strength drop [12]. The secondary fine particles are also suitable for the tensile and burst index [10]. Salehi et al. [18] reported the maximum specific energy that the bagasse pulp tolerates before the strength start deterioration is 163 kwh/t. Unlike bagasse, the strength development in the Enset fiber pulp showed a potential increment beyond a refining energy of 250 kwh/t.

The higher tensile strength in the pulp produced by 16% NaOH can also be correlated with its high xylose content. Omer et al. [23] reported the good bonding ability of the fiber with high hemicellulose content. High hemicellulose content in the pulp makes the fiber more flexible and fibrillated during refining [12,19]. As reported by Olejnik et al. [5], the tensile strength of paper from eucalyptus tends to be constant after the refining energy of 200 kwh/t. However, in the case of Enset fiber pulp, the tensile index still has the potential to increase if the refining energy increases beyond 250 kwh/t.

The tear index dropped with refining because it depends on the individual fiber length, strength, degree of inter-fiber bonding, and fiber orientation in the paper (Figure 6d; [7]). The tear index is lower when the fibers are short, weak, and poorly bonded. It is more dependent on fiber breakage than bond breakage. Longer and stronger fibers provide higher tear strength because of the work needed to pull the fibers from the sheet [24].

The porosity and specific volume of the sheet decrease with SR° because when the fibers are more flexible, denser papers are produced [7,18]. In addition, the CWT and fiber width reduction make the paper more compact each other and result in lower specific volume. The increasing ratio of small non-fiber fragments and fine particles fills the paper and reduces porosity [10].

Uncorrelated changes were clearly observed because the paper-making pulp was a highly heterogeneous lignocellulosic material, and there was a wide range of fiber length distribution in the pulp compared to pulp from other annual plants, as shown in Figure 6 [20,25]. As a result, no satisfactory mathematical equations unambiguously predict pulp properties based on pulping and refining parameters [5].

## 3. Materials and Methods

### 3.1. Raw Material Characterization

Enset fiber bundles were collected from southwestern Ethiopia, Wolkite, and Wolayita. The samples were air-dried and cut into 2–5 cm-long pieces. Extensive chemical composition analysis was conducted in our previous study regarding extractive content, ash content, carbohydrate composition, and lignin content [26]. The chemical compositions of the Enset fiber are summarized in Table 2.

### 3.2. Soda Pulping of the Enset Fiber

A total of 300 g (oven-dry) of Enset fiber was used for soda pulping in a 15-Liter laboratory rotating digester at a maximum temperature of 160 °C, 170 °C, and 180 °C. Sodium hydroxide charges were 16%, 20%, and 24% based on the oven-dry weight of the raw material. The solid-to-liquid ratio was kept at 1:4 for all experiments. The maximum temperature was attained within 60 min and kept there for another 60 min.

Three pulping conditions were selected for large-scale pulping in an 80-Liter, steam-heated horizontal reactor with a mixer rotated at 0.12–0.13 rpm. The pulping was performed at 170 °C and at NaOH charges of 16%, 20%, and 24%.

The resulting pulps from both reactors were washed and dewatered by centrifugation in a spinner (Thomas Centri 776 SEK, Thomas, Neunkirchen, Germany) for 10 min at 2800 rpm. The pulp yield was analyzed after homogenizing of the pulp for 10 min in a rotary stirrer (Hobart, Offenburg, Germany) and reported as a percentage of the initial weight of fiber.

### 3.3. Chemical Composition of the Enset Fiber Soda Pulp

A 200 mg soda pulp sample was air-dried and milled with a disc mill (SM 200, Retsch, Haan, Germany) to determine the monomeric carbohydrate composition from the two-stage acid hydrolysis filtrates by Borate-AEC using a Dionex Ultimate 3000 (Dionex, Sunnyvale, CA, USA) as described in Lorenz et al. [27]. Specifically, 200 mg of oven-dry material were hydrolyzed with 2 mL of 72% sulfuric acid at 30 °C for exactly one hour to break up the crystalline structure of the cellulose. Thereafter, the sulfuric acid was diluted by adding 56 mL of water, and then the samples were put in an autoclave at 120 °C and 1.2 bar for 40 min to degrade the remaining oligosaccharide chains into monomeric sugars. After cooling, the samples were filtered on a N°4-sintered glass crucible. The hydrolysis residue (HR) was oven-dried and gravimetrically determined, and it represented the acid-insoluble lignin content, which is analogous to Klason lignin. The quantitative and qualitative carbohydrate composition in the hydrolysates was analyzed by borate-HPAEC analysis. Dionex Ultimate 3000 and an anion exchange resin MCL Gel CA08F (Mitsubishi Chemical, Tokyo, Japan), in a column with the dimensions of 5 × 120 mm (Omnifit) (packed at 65 °C), were used for chromatographic separation. The mobile phase consisted of two potassium tetraborate/boric acid buffers in water: 0.3 M (A; pH 8.6) and 0.9 M (B; pH 9.5). Separation was performed at 65 °C with a flow rate of 0.7 mL/min. The elution program started with 90% A and 10% B and changed to 10% A and 90% B within 35 min. This rate was kept constant for 8 min and changed back to 90% A and 10% B within 7 min. Prior to detection, a post-column derivatization by Cubicinchoninate (0.35 mL/min) was applied at 105 °C in a Teflon^®^ coil of 30 m length and 0.3 mm diameter. The analytes were detected at 560 nm via UV/VIS-detection. The contents of the carbohydrates and lignin were determined as triplicates. The standard deviation (SD) was between 0.2% and 1.7% for the carbohydrate content and less than 0.5% for the lignin content (HR).

The UV-Spectrophotometer LAMBDA 650 (PerkinElmer, Waltham, MA, USA) at a wavelength of 205 nm was used to determine the acid-soluble lignin content according to Maekawa et al. [28]. The Klason lignin content was calculated from the solid residue [26]. Kappa numbers were determined using the TAPPI standard T236 om-99 (1999) [29].

### 3.4. Pulp Beating and Refining

The never dried pulp from the 15-Liter rotating digester was beaten using a Jokro mill (FRANK-PTI, Birkenau, Germany) according to the German standard Zellcheming V/5/60 (1960) [30]. The mechanical action was conducted between the inner and outer walls of the mill at a principle of centrifugal force of 150 rpm for 4 min, 20 min, and 30 min. The fiber morphology and sheet paper strength were measured before and after beating.

The pulps from the large-scale reactor were subjected to refining using a fully automated laboratory refiner (Votih LR 40, FRANK-PTI) with disc refiner headsets (standard plate for long fibers). The full refiner was operated under a refining intensity of 1 J/m and a rotation speed of 2000 rpm. The refining energy range was from 0 kwh/t to 250 kwh/t. A pulp consistency of 3% was kept for each batch of samples.

After beating or refining, the pulp was disintegrated using a standard pulp disintegrator based on ISO 5263-2:2004 (2004) [31] to disperse the pulp fiber into the solution. The beating degree was determined with a Schopper-Riegler (°SR) freeness tester type SR1 (Karl Schröder KG, Weinheim, Germany). The resulting pulp with different degrees of refining was analyzed using a Kajani Fiber lab (Metso, Helsinki, Finland) to determine the fiber length distribution and fiber morphology characterization, such as the average fiber length, the amount of fine material, fiber width, and the fiber curl and kink index.

### 3.5. Analysis of the Paper Strength

Laboratory handsheets of about 80 g/m^2^ basis weight were prepared using a Rapid-Koethen sheetformer (FRANK-PTI, Birkenau, Germany). The resulting paper was acclimatized at 23 ± 1 °C and 55 ± 2% relative humidity overnight before testing its strength properties.

The compression, burst, tensile, and tear strength were measured according to DIN 54518:2004 (2004) [32], ISO 2759:2014 (2014) [33], ISO 1924-2:2009 (2009) [34], and ISO 1974:2012-09 (2012) [35], respectively, using instruments manufactured by FRANK-PTI GmbH (Birkenau, Germany). The results were normalized by dividing with the grammage of each paper sheet.

## 4. Conclusions

The Enset fiber soda pulp shows superior quality for the paper industry compared to other annual plants and agricultural residues. It is a better suited raw material compared to wheat straw and bagasse. The soda pulp from Enset fiber was effectively refined by a Voith LR 40 refiner, and the strength of the paper was improved.

One main new achievement was the observation that the beating of Enset fiber pulp in a Jokro mill had almost no effect on the fiber morphology and the development of paper strength. This put emphasis on the fact that the mechanisms in Jokro mill and laboratory refiner beating are completely different and therefore have a different impact on the fiber. It was found that fibers are more squeezed and maybe crushed in Jokro mill beating, while the fibers are actually cut in laboratory refining. This can be seen in Figure 5a,b, where the fraction of “long” fibers (3.2–7.6 mm) decreased from >20% to less than 10% after refining. Only the cutting of fibers in laboratory refining led to a change in fiber and paper properties, while the squeezing and crushing in Jokro mill beating had almost no effect on fiber and paper properties.

Based on this result, the fiber can be used for packaging or sack paper due to its high tensile strength and curl index. Due to its porosity, Enset fiber pulp can also be used for specialty paper, such as tea bags or as filter paper. Sanitary purposes are also another possibility because of its high-water permeability due to higher hemicellulose content. Depending on the intended product being made, there should be always a trade-off between various properties during pulping and refining stages.

## Figures and Tables

**Table 1:** Fiber morphology of the Enset soda pulp prepared at 160 °C and 16% NaOH using the rotating digester and Jokro mill beating.

Beating Time (min)	Fiber Length (mm)	Fiber Width (µm)	CWT	Fines Fraction (%)	Fibrillation (%)
0	2.8	17.5	7.0	5.0	9.7
4	2.8	17.7	6.4	5.0	9.6
20	2.8	17.7	6.1	5.7	9.7
30	2.8	17.7	6.6	5.4	9.6

**Table 2:** Summary of the chemical compositions of the Enset fiber [27].

**Extractives** (**%**)	Petrol ether	0.1
	Acetone	0.9
	Water	3.4
	**∑**	**4.4**
**Lignin** (**%**)	Klason lignin	10.8
	Acid-soluble	2.5
	**∑**	**13.3**
**Monosaccharides** (**%**)	Rhamnose, mannose, arabinose, galactose	2.4
	Xylose	10.7
	Glucose	58.9
	**∑**	**72.1**
**Ash** (**%**)	Silica	2.6
	**∑**	**5.4**

## Data Availability

The data presented in this study are available from the corresponding authors upon request.

## Supplementary Materials

The following supporting information can be downloaded at: https://www.mdpi.com/article/10.3390/molecules29204874/s1. Figure S1: Selected physical paper properties of pulps from laboratory-scale pulping with a 15 L rotary digester at different temperatures and NaOH charges; Figure S2: Process scheme—from the Enset plant to Enset fibers to Enset paper grade pulp; Figure S3: Correlation between Xylose content and beating degree of unrefined pulp at different NaOH charges (large-scale pulping at 170 °C); Figure S4: Example Chromatogram from carbohydrate analysis via borate-HPAEC analysis; Table S1: Analysis of variance (Anova) of the pulping data (pulp yield and kappa number) at laboratory scale with a rotary digester; Table S2: Analysis of variance (Anova) of fiber morphology data, and the proportion of different fiber fractions after pulping at laboratory scale with a rotary digester.
