# Supplementary material for: Production of Long-Fiber Pulp from Enset Plant Residues by Soda Pulping
Source: Molecules. 2024 Oct 14;29(20):4874. doi: 10.3390/molecules29204874 (PMC11510142; doi:10.3390/molecules29204874)
Supplement: Supplementary file 1 [file molecules-29-04874-s001.zip › Figure S1.pdf]

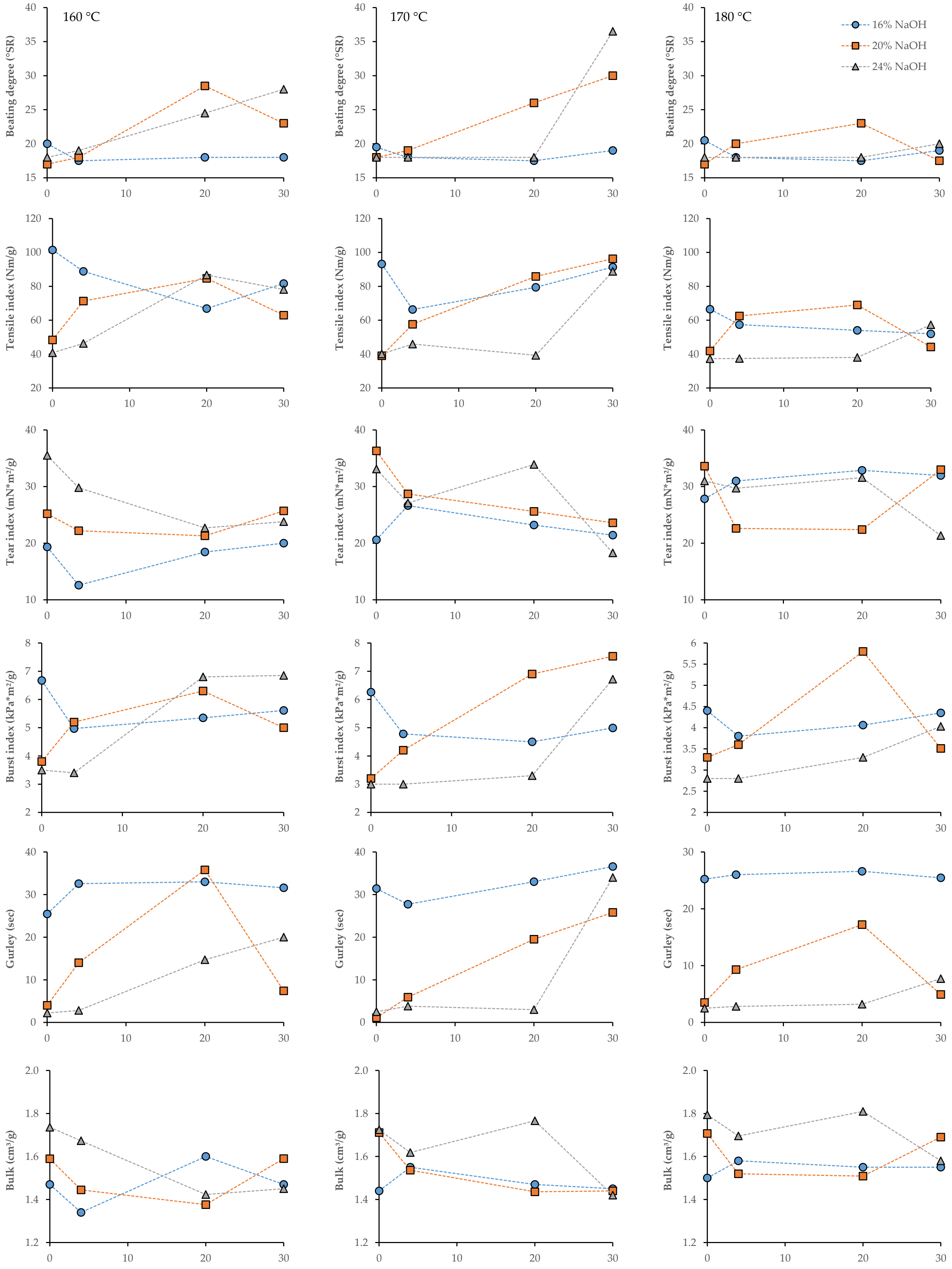

Figure S1. Selected physical paper properties of pulps from laboratory-scale pulping with 15 L rotary digester at different temperatures and NaOH charges.
