# Supplementary material for: Production of Long-Fiber Pulp from Enset Plant Residues by Soda Pulping
Source: Molecules. 2024 Oct 14;29(20):4874. doi: 10.3390/molecules29204874 (PMC11510142; doi:10.3390/molecules29204874)
Supplement: Supplementary file 1 [file molecules-29-04874-s001.zip › Figure S3.pdf]

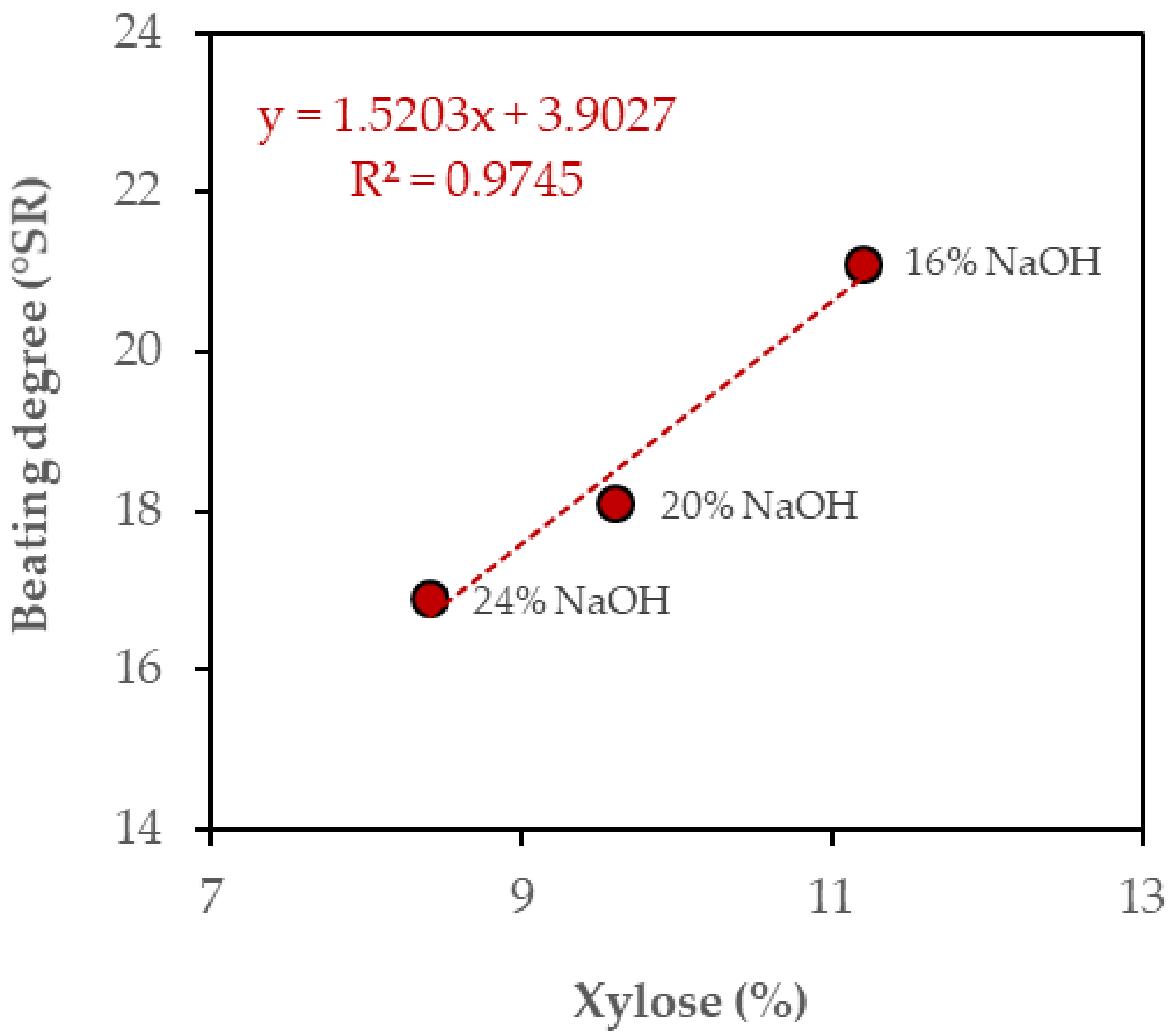


**Figure S3.** Correlation between Xylose content and beating degree of unrefined pulp at different NaOH charges (large-scale pulping at 170 °C).
