# Supplementary material for: Production of Long-Fiber Pulp from Enset Plant Residues by Soda Pulping
Source: Molecules. 2024 Oct 14;29(20):4874. doi: 10.3390/molecules29204874 (PMC11510142; doi:10.3390/molecules29204874)

Sample Analysis Report

Soda pulp 160 °C 24% NaOH (1), Hy.Rü.: 1,5%

**Sample Name:**	Fiete, Q 791	**Sample No.:**	75
**Sequence Name:**	050824		
**Program Method:**	Borat	**Injection vol.:**	10.0
**Quantitation Method:**	Borat - testing	**Dilution Factor:**	20.0000
**Date Time Collected:**	06.06.2024 4:04 AM	**Sample Wt.:**	198.6000
**System Operator:**	n.a.	**Sample Amt.:**	1.0000

Peak No.	Component Name	Retention Time	Area mAU*min	Height mAU	Amount
1	Cellobiose	12.82	12.65399	16.61087	0.87
2		13.33	3.61318	7.69935	n.a.
3	Rhamnose	15.77	0.31027	0.51797	0.02
4	Mannose	22.22	2.41922	2.52403	0.20
5		23.21	1.72080	2.32688	n.a.
6	Arabinose	25.67	2.16720	1.22711	0.29
7	Galaktose	28.02	1.41308	1.22523	0.13
8	Xylose	29.32	68.52013	57.91427	9.46
9	Glucose	32.82	620.53895	480.71665	81.97

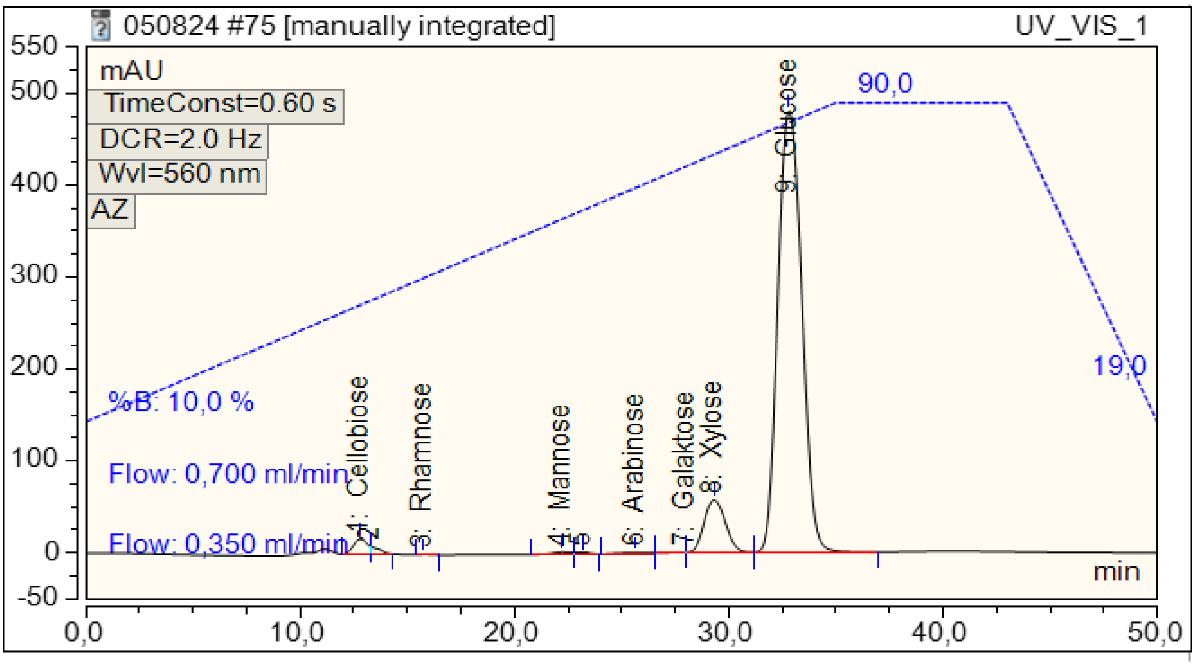

Supplement: Supplementary file 1 [file molecules-29-04874-s001.zip › Figure S4.pdf]
