# Supplementary material for: Production of Long-Fiber Pulp from Enset Plant Residues by Soda Pulping
Source: Molecules. 2024 Oct 14;29(20):4874. doi: 10.3390/molecules29204874 (PMC11510142; doi:10.3390/molecules29204874)
Supplement: Supplementary file 1 [file molecules-29-04874-s001.zip › Table S1.pdf]

Table S1. Analysis of Variance (Anova) of pulping data (pulp yield and kappa number) at laboratory scale with rotary digester.

Pulp Yield (%)

	160°C	170°C	180°C
16% NaOH	63.3	63.8	61.4
20% NaOH	60.6	59.5	57.0
24% NaOH	60.0	54.8	54.0

Anova: Two-Factor Without Replication

SUMMARY	*Count*	*Sum*	*Average*	*Variance*
Row 1	3	188.4	62.8	1.6
Row 2	3	177.1	59.0	3.3
Row 3	3	168.7	56.2	10.5
Column 1	3	183.8	61.3	3.2
Column 2	3	178.0	59.3	20.1
Column 3	3	172.3	57.4	13.8

ANOVA

Source of Variation	*SS*	*df*	*MS*	*F*	***P-value***	*F crit*
Rows	65.39	2	32.69	14.6814	**0.01**	6.94
Columns	22.00	2	11.00	4.9404	**0.08**	6.94
Error	8.91	4	2.23			
Total	96.30	8				

Kappa number

	160°C	170°C	180°C
16% NaOH	15.6	16.6	15.8
20% NaOH	14.1	10.8	7.8
24% NaOH	9.8	8.4	2.8

Anova: Two-Factor Without Replication

SUMMARY	*Count*	*Sum*	*Average*	*Variance*
Row 1	3	48.0	16.0	0.3
Row 2	3	32.7	10.9	10.1
Row 3	3	21.0	7.0	13.7
Column 1	3	39.5	13.2	9.1
Column 2	3	35.8	11.9	17.8
Column 3	3	26.4	8.8	43.1

ANOVA

Source of Variation	*SS*	*df*	*MS*	*F*	***P-value***	*F crit*
Rows	122.26	2	61.13	13.9611	**0.02**	6.94
Columns	30.66	2	15.33	3.5008	**0.13**	6.94
Error	17.51	4	4.38			
Total	170.43	8				
