# Supplementary material for: Production of Long-Fiber Pulp from Enset Plant Residues by Soda Pulping
Source: Molecules. 2024 Oct 14;29(20):4874. doi: 10.3390/molecules29204874 (PMC11510142; doi:10.3390/molecules29204874)
Supplement: Supplementary file 1 [file molecules-29-04874-s001.zip › Table S2.pdf]

Table S2. Analysis of Variance (Anova) of fibre morphology data; Proportion of different fibre fractions after pulping at laboratory scale with rotary digester.

Proportion of fibre fraction (%): 3.2 - 7.6 mm

	Beating time (min)			
	0 Min	4 Min	20 Min	30 min
160 °C	33.58	29.75	31.83	30.08
	32.83	33.67	31.20	34.02
170 °C	32.20	31.48	32.07	32.53
	32.29	32.02	33.49	33.17
180 °C	25.38	24.74	24.80	26.07
	25.25	24.45	25.31	26.64

Anova: Two-Factor With Replication

SUMMARY	0 Min	4 Min	20 Min	30 min	Total
160 °C					
Count	2	2	2	2	8
Sum	66.41	63.42	63.03	64.10	256.96
Average	33.21	31.71	31.52	32.05	32.12
Variance	0.28	7.68	0.20	7.76	2.77
170 °C					
Count	2	2	2	2	8
Sum	64.49	63.50	65.56	65.70	259.25
Average	32.25	31.75	32.78	32.85	32.41
Variance	0.00	0.15	1.01	0.20	0.42
180 °C					
Count	2	2	2	2	8
Sum	50.63	49.19	50.11	52.71	202.64
Average	25.32	24.60	25.06	26.36	25.33
Variance	0.01	0.04	0.13	0.16	0.53
Total					
Count	6	6	6	6	
Sum	181.53	176.11	178.70	182.51	
Average	30.26	29.35	29.78	30.42	
Variance	14.89	15.15	14.00	11.66	

ANOVA

Source of Variation	*SS*	*df*	*MS*	*F*	***P-value***	*F crit*
Sample	256.69	2.00	128.35	87.36	**0.00**	3.89
Columns	4.19	3.00	1.40	0.95	**0.45**	3.49
Interaction	4.16	6.00	0.69	0.47	**0.82**	3.00
Within	17.63	12.00	1.47			
Total	282.67	23.00				

Proportion of fibre fraction (%): 2.0 - 3.2 mm

	Beating time (min)			
	0 Min	4 Min	20 Min	30 min
160 °C	38.81	38.41	37.96	39.42
	38.47	37.95	38.92	38.06
170 °C	40.80	40.25	40.31	41.26
	40.76	40.51	39.22	40.57
180 °C	37.60	37.35	37.88	38.36
	38.49	36.87	37.64	38.88

Anova: Two-Factor With Replication

SUMMARY	0 Min	4 Min	20 Min	30 min	Total
160 °C					
Count	2	2	2	2	8
Sum	77.28	76.36	76.88	77.48	308.00
Average	38.64	38.18	38.44	38.74	38.50
Variance	0.06	0.11	0.46	0.92	0.27
170 °C					
Count	2	2	2	2	8
Sum	81.56	80.76	79.53	81.83	323.68
Average	40.78	40.38	39.77	40.92	40.46
Variance	0.00	0.03	0.59	0.24	0.35
180 °C					
Count	2	2	2	2	8
Sum	76.09	74.22	75.52	77.24	303.07
Average	38.05	37.11	37.76	38.62	37.88
Variance	0.40	0.12	0.03	0.14	0.43
Total					
Count	6	6	6	6	
Sum	234.93	231.34	231.93	236.55	
Average	39.16	38.56	38.66	39.43	
Variance	1.75	2.27	1.05	1.59	

ANOVA

Source of Variation	*SS*	*df*	*MS*	*F*	***P-value***	*F crit*
Sample	28.96	2.00	14.48	56.20	**0.00**	3.89
Columns	3.06	3.00	1.02	3.95	**0.04**	3.49
Interaction	1.27	6.00	0.21	0.82	**0.57**	3.00
Within	3.09	12.00	0.26			
Total	36.38	23.00				

Proportion of fibre fraction (%): 1.2 - 2.0 mm

	Beating time (min)			
	0 Min	4 Min	20 Min	30 min
160 °C	19.15	21.95	20.76	21.19
	20.00	19.82	20.68	19.54
170 °C	19.10	19.57	18.85	18.52
	18.67	18.86	18.98	17.84
180 °C	24.28	24.83	24.33	23.82
	24.15	25.14	24.63	22.98

Anova: Two-Factor With Replication

SUMMARY	0 Min	4 Min	20 Min	30 min	Total
160 °C					
Count	2	2	2	2	8
Sum	39.15	41.77	41.44	40.73	163.09
Average	19.58	20.89	20.72	20.37	20.39
Variance	0.36	2.27	0.00	1.36	0.86
170 °C					
Count	2	2	2	2	8
Sum	37.77	38.43	37.83	36.36	150.39
Average	18.89	19.22	18.92	18.18	18.80
Variance	0.09	0.25	0.01	0.23	0.25
180 °C					
Count	2	2	2	2	8
Sum	48.43	49.97	48.96	46.80	194.16
Average	24.22	24.99	24.48	23.40	24.27
Variance	0.01	0.05	0.05	0.35	0.44
Total					
Count	6	6	6	6	
Sum	125.35	130.17	128.23	123.89	
Average	20.89	21.70	21.37	20.65	
Variance	6.81	7.57	6.46	5.89	

ANOVA

Source of Variation	*SS*	*df*	*MS*	*F*	***P-value***	*F crit*
Sample	126.77	2.00	63.38	151.14	**0.00**	3.89
Columns	3.99	3.00	1.33	3.17	**0.06**	3.49
Interaction	1.83	6.00	0.31	0.73	**0.64**	3.00
Within	5.03	12.00	0.42			
Total	137.62	23.00				

Proportion of fibre fraction (%): **0.5 - 1.2 mm**

	Beating time (min)			
	0 Min	4 Min	20 Min	30 min
160 °C	7.04	8.13	7.79	7.63
	7.13	7.05	7.56	6.92
170 °C	6.44	7.09	7.05	6.24
	6.75	7.00	6.71	6.94
180 °C	10.68	10.92	10.75	9.85
	10.15	11.29	10.39	9.56

Anova: Two-Factor With Replication

SUMMARY	0 Min	4 Min	20 Min	30 min	Total
160 °C					
Count	2	2	2	2	8
Sum	14.17	15.18	15.35	14.55	59.25
Average	7.09	7.59	7.68	7.28	7.41
Variance	0.00	0.58	0.03	0.25	0.19
170 °C					
Count	2	2	2	2	8
Sum	13.19	14.09	13.76	13.18	54.22
Average	6.60	7.05	6.88	6.59	6.78
Variance	0.05	0.00	0.06	0.25	0.09
180 °C					
Count	2	2	2	2	8
Sum	20.83	22.21	21.14	19.41	83.59
Average	10.42	11.11	10.57	9.71	10.45
Variance	0.14	0.07	0.06	0.04	0.33
Total					
Count	6	6	6	6	
Sum	48.19	51.48	50.25	47.14	
Average	8.03	8.58	8.38	7.86	
Variance	3.49	4.02	3.05	2.25	

ANOVA

Source of Variation	*SS*	*df*	*MS*	*F*	***P-value***	*F crit*
Sample	61.68	2.00	30.84	240.88	**0.00**	3.89
Columns	1.92	3.00	0.64	5.01	**0.02**	3.49
Interaction	0.83	6.00	0.14	1.08	**0.43**	3.00
Within	1.54	12.00	0.13			
Total	65.97	23.00				

Proportion of fibre fraction (%): **0.2 - 0.5 mm**

	Beating time (min)			
	0 Min	4 Min	20 Min	30 min
160 °C	1.19	1.46	1.41	1.40
	1.33	1.21	1.36	1.25
170 °C	1.23	1.36	1.44	1.23
	1.30	1.35	1.34	1.23
180 °C	1.77	1.85	1.92	1.61
	1.69	1.97	1.73	1.67

Anova: Two-Factor With Replication

SUMMARY	0 Min	4 Min	20 Min	30 min	Total
160 °C					
Count	2	2	2	2	8
Sum	2.52	2.67	2.77	2.65	10.61
Average	1.26	1.34	1.39	1.33	1.33
Variance	0.01	0.03	0.00	0.01	0.01
170 °C					
Count	2	2	2	2	8
Sum	2.53	2.71	2.78	2.46	10.48
Average	1.27	1.36	1.39	1.23	1.31
Variance	0.00	0.00	0.00	0.00	0.01
180 °C					
Count	2	2	2	2	8
Sum	3.46	3.82	3.65	3.28	14.21
Average	1.73	1.91	1.83	1.64	1.78
Variance	0.00	0.01	0.02	0.00	0.02
Total					
Count	6	6	6	6	
Sum	8.51	9.20	9.20	8.39	
Average	1.42	1.53	1.53	1.40	
Variance	0.06	0.09	0.06	0.04	

ANOVA

Source of Variation	*SS*	*df*	*MS*	*F*	***P-value***	*F crit*
Sample	1.12	2.00	0.56	73.63	**0.00**	3.89
Columns	0.09	3.00	0.03	4.16	**0.03**	3.49
Interaction	0.04	6.00	0.01	0.80	**0.59**	3.00
Within	0.09	12.00	0.01			
Total	1.34	23.00				

Proportion of fibre fraction (%): **< 0.2 mm**

	Beating time (min)			
	0 Min	4 Min	20 Min	30 min
160 °C	0.23	0.26	0.25	0.26
	0.23	0.24	0.25	0.20
170 °C	0.23	0.23	0.25	0.23
	0.21	0.22	0.26	0.26
180 °C	0.28	0.30	0.31	0.26
	0.27	0.28	0.30	0.28

Anova: Two-Factor With Replication

SUMMARY	0 Min	4 Min	20 Min	30 min	Total
160 °C					
Count	2	2	2	2	8
Sum	0.46	0.50	0.50	0.46	1.92
Average	0.23	0.25	0.25	0.23	0.24
Variance	0.00	0.00	0.00	0.00	0.00
170 °C					
Count	2	2	2	2	8
Sum	0.44	0.45	0.51	0.49	1.89
Average	0.22	0.23	0.26	0.25	0.24
Variance	0.00	0.00	0.00	0.00	0.00
180 °C					
Count	2	2	2	2	8
Sum	0.55	0.58	0.61	0.54	2.28
Average	0.28	0.29	0.31	0.27	0.29
Variance	0.00	0.00	0.00	0.00	0.00
Total					
Count	6	6	6	6	
Sum	1.45	1.53	1.62	1.49	
Average	0.24	0.26	0.27	0.25	
Variance	0.00	0.00	0.00	0.00	

ANOVA

Source of Variation	*SS*	*df*	*MS*	*F*	***P-value***	*F crit*
Sample	0.01	2.00	0.01	21.74	**0.00**	3.89
Columns	0.00	3.00	0.00	3.26	**0.06**	3.49
Interaction	0.00	6.00	0.00	0.79	**0.59**	3.00
Within	0.00	12.00	0.00			
Total	0.02	23.00				
