# Supplementary figures and images for: Production of Long-Fiber Pulp from Enset Plant Residues by Soda Pulping
Source: Molecules. 2024 Oct 14;29(20):4874. doi: 10.3390/molecules29204874 (PMC11510142; doi:10.3390/molecules29204874)

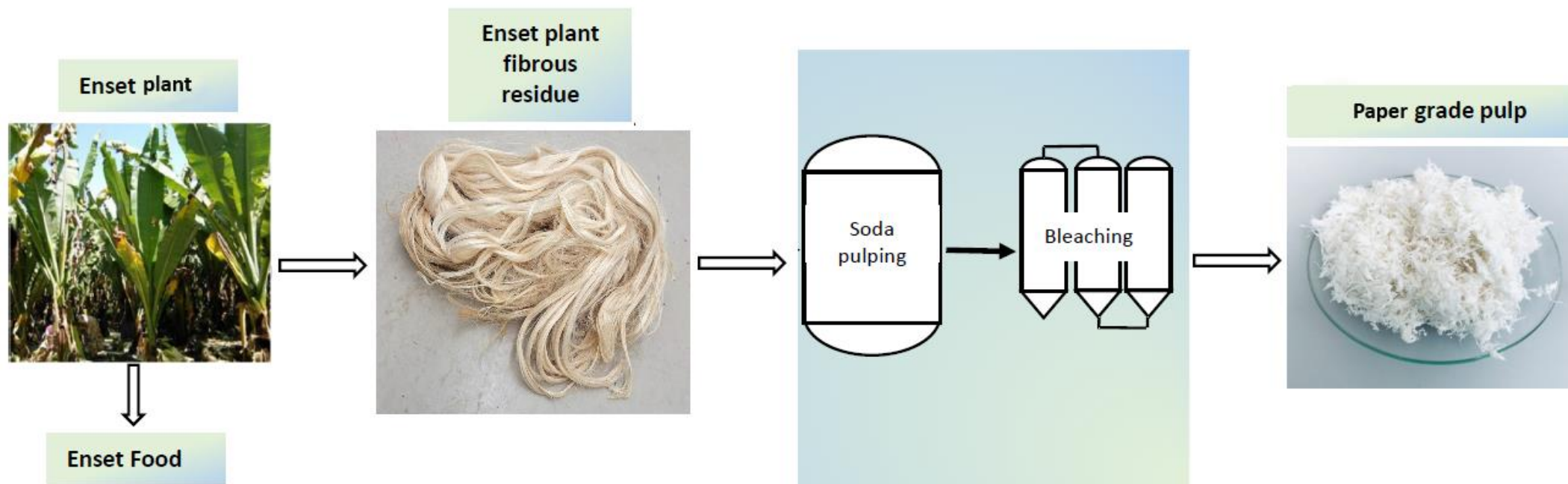

**Figure S2.** Process scheme – from the Enset plant to Enset fibers to Enset paper grade pulp.

Supplement: Supplementary file 1 [file molecules-29-04874-s001.zip › Figure S2.pdf]
